# MRI-Based Morphometric Comparison of Lower Leg Muscles and Tendons in Individuals With Medial Tibial Stress Syndrome

**DOI:** 10.1155/bmri/8827692

**Published:** 2024-12-16

**Authors:** Lucas Nogueira de Oliveira, João Luiz Quagliotti Durigan, Caroline Ribeiro Sanchez, Henrique Mansur, Andressa Beatriz Beltrão Rosa, Rita de Cássia Marqueti

**Affiliations:** ^1^Orthopaedic and Trauma Unit, Hospital de Base do Distrito Federal, Brasília, Distrito Federal, Brazil; ^2^Laboratory of Molecular Analysis, Graduate Program in Rehabilitation Science, Faculdade de Ciências da Saúde e Tecnologias, Universidade de Brasília, Brasília, Distrito Federal, Brazil; ^3^Laboratory of Muscle and Tendon Plasticity, Graduate Program in Rehabilitation Science, Faculdade de Ciências da Saúde e Tecnologias, Universidade de Brasília, Brasília, Distrito Federal, Brazil; ^4^Musculoskeletal Group, Villas-Boas Clinic, Brasília, Distrito Federal, Brazil; ^5^Orthopaedic Group, DF Star Hospital-Rede D'or, Brasília, Distrito Federal, Brazil

**Keywords:** deep posterior compartment, flexor hallucis longus, MRI, MTSS, muscle volume

## Abstract

Runners frequently suffer from medial tibial stress syndrome (MTSS), often linked to excessive eccentric muscle contractions causing periosteal traction by the muscles in the deep posterior compartment. However, the effects of MTSS on these muscles and tendons remain underexplored. This study is aimed at investigating changes in muscle and tendon volumes in this compartment, as well as cross-sectional area measurements, using magnetic resonance imaging. Thirty individuals were divided into two groups: MTSS (*n* = 18; mean age 30.3 ± 12.4) and control (*n* = 12; age 35.2 ± 9.2). The anterior, deep posterior, superficial posterior, and lateral compartment muscles, along with their respective tendons, were compared between groups, and possible sex differences were also evaluated. The deep posterior compartment showed a significant volume difference of 0.41 cm^3^/kg^3^/^4^ in the MTSS group (*p* = 0.034), primarily due to the flexor hallucis longus (FHL), which had a 0.55 cm^3^/kg^3^/^4^ greater normalized volume (17.12% greater mean muscle volume) compared to controls (*p* = 0.023; Cohen *d* = 0.895). No association between sex and MTSS was found (*p* = 0.752). In conclusion, the FHL muscle exhibited increased normalized volume in the MTSS group compared to controls, with no sex-related differences in MTSS. Clinicians should consider the assessment of FHL muscle volume in routine evaluations of patients presenting with symptoms suggestive of MTSS.

## 1. Introduction

Running has become increasingly popular due to its beneficial health effects [1, 2]. Consequently, the number of injuries associated with this exercise method has increased substantially among recreational and professional athletes [3]. Nearly 15% of all injuries described in sports medicine are related to tibial pain, over 60% of all pain in athletes occurs in the lower limbs, and about 55% of runners experience an injury at least once per year [4, 5]. One of the most frequent injuries is medial tibial stress syndrome (MTSS), which has a cause and effect relationship with running activities [6, 7]. It has an incidence of between 4% and 35% in the athletic and military population [8, 9]. MTSS is diagnosed by palpation of the posteromedial tibial border (PTB) that elicits pain or when an individual reports exercised-induced pain in the distal two-thirds of the PTB, both of which are associated with a magnetic resonance imaging (MRI) exam describing periosteal edema or stress fractures [7, 10–13]. However, some authors claimed that stress fractures are different entities and there is no natural evolution from MTSS to stress fractures [14–17]. Risk factors for MTSS also include the feminine gender, age, and patterns of lower limb kinematics (greater flexed hip external rotation in males and positive navicular drop test) [6, 18–20].

The main hypotheses for the causes of MTSS focus on tibial microfractures from repetitive movements (bone overload injury), muscle weakness leading to inadequate impact absorption, and excessive eccentric muscle contraction, which may result in periosteal traction and, consequently, regional inflammatory pain [9, 21–23]. Biomechanical aspects of the running gait related to the lower leg deep posterior compartment (DPC) muscles were demonstrated as a possible cause of tenting effect at the distal tibial fascia, which leads to increased tension at its posteromedial insertions [24]. Also, one study reported that there is increased maximal voluntary isometric contraction torque for plantar flexion of the first metatarsophalangeal joint related to extrinsic deep flexors of the foot in patients with MTSS [25]. The etiopathology of MTSS remains unknown, although some partially accepted theories exist. The tenting effect theory may contribute to understanding the role of tibial fascia traction in the origin of pain in MTSS, as it has been hypothesized that its etiopathology could be similar to that of plantar fasciitis [24]. Nevertheless, there is still a knowledge gap regarding how the morphological aspects of leg muscles behave in vivo to create the tenting effect. Identifying the exact risk factors for the development of MTSS is essential for potentially improving the future diagnosis and management of this condition [26].

MRI exam is considered the gold standard for diagnosing MTSS [13, 27], since it can assess the axial cross-sectional areas of muscles and tendons through manual segmentation techniques [28]. Changes in DPC muscles and tendons may further inform the impact of morphological alterations on the tenting effect theory at the posteromedial tibial fascia insertion in individuals with MTSS, as observed using MRI. This study hypothesizes that individuals with MTSS exhibit increased volume and cross-sectional area in the DPC muscles, particularly the flexor hallucis longus (FHL), which may contribute to a “tenting effect” on the tibial fascia associated with MTSS-related pain. The purpose of this study was, therefore, to investigate the changes associated with MTSS in DPC muscle volumes and axial cross-sectional areas measurements and to compare these findings to a control group, all assessed through MRI.

## 2. Materials and Methods

### 2.1. Study Design

This is a retrospective, observational, and cross-sectional study. All participants provided their informed consent prior to the MRI exam. The study was approved by the Institutional Review Board at the Universidade de Brasília (Protocol 54414121.0.0000.8093). Data were collected retrospectively from a database of MRI exams and pre-exam questionnaires in a private radiology clinic of the Brazil Federal District ranging from 2018 to 2022.

### 2.2. Participants

Eighteen participants with MTSS were enrolled in this study after applying the inclusion and exclusion criteria based on the pre-exam questionnaires. The inclusion criteria included participants aged between 15 and 50 years with a diagnosis of MTSS. Patients with a history of previous lower limb surgery, pregnancy, lower leg fractures, recent trauma, neuromuscular disorders, or undergoing systemic chronic disease treatment were excluded. A control group was formed with a convenience sample of the 20 most recent MRI exams categorized as “normal,” rather than “MTSS,” with normal descriptive MRI reports of the lower leg made by two senior radiologists. After investigating the pre-exam questionnaires, only 12 participants were eligible for statistical analysis, as the others reported pain in the posterior and medial regions of the lower leg. Therefore, participants were grouped based on MTSS diagnosis and the control groups.

### 2.3. MRI Acquisition

All MRIs were performed to determine the presence of MTSS, and the axial cross-sectional areas of the lower leg muscles and tendons were obtained along their length (PHILIPS Ingenia 3 Tesla HP, Amsterdam, Netherlands). The scanning time lasted approximately 30 min per patient. Axial, coronal, and sagittal sequences were performed for all exams. Participants lay supine with their hips and knees extended and the ankles fixed in a relaxed position. The selected sequence for analysis was the turbo spin echo axial T1-weighted sequence, considered the best anatomical sequence [29]. The protocol had an acquisition time of 190.75 s, repetition time (TR) of 755, time to echo (TE) of 13.5, matrix of 364 × 280, field of view (FOV) of 400 × 200 mm, voxel size of 0.4 × 0.4, a thickness of 6 mm, and a cutting range of 0.4. An eight-channel receive-only multicoil was used as a receiver. Imaging was obtained continuously from the distal third of the femur to the lowest aspect of the ankle. All included participants were examined using the same image acquisition technique on one of two devices with identical specifications.

### 2.4. Segmentation Technique

The segmented muscles were tibialis anterior, extensor digitorum longus, extensor hallucis longus, tibialis posterior, flexor digitorum longus (FDL), FHL, fibularis (FL) (longus and brevis cojoined in one measure), soleus, and gastrocnemius (both heads in one measure). The segmented tendons were tibialis posterior, FDL, FHL, FL (longus and brevis cojoined in one measure), and Achilles tendon. The muscle length was segmented in the axial T1-weighted turbo spin-echo image using the software OsiriX (Pixmeo SARL, version 2.5.1, Switzerland). Some authors described methods with reduced segmentation of slices to estimate the total muscle volume and reported the accuracy of the muscle volume estimates [29–34]. Reports mentioned that slices ranging from 5 to 9 mm of thickness with a 1.0- to 3.1-cm distance interval between slices measured could provide adequate precision to muscle volume estimations [31, 32, 35–37].

**Figure 1:**
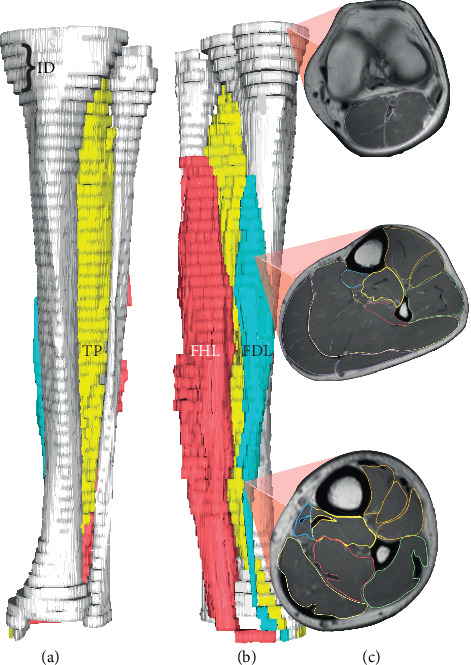
3-D magnetic resonance rendered image and segmented slices of a MTSS group participant. (a) Anterior view, (b) posteromedial view, and (c) slice axial cross-sectional areas. ID, interslice space of 2.4 cm; FHL, flexor hallucis longus; FDL, flexor digitorum longus; TP, tibialis posterior.

The axial cross-sectional area records were obtained in an interval of 2.4 cm between slices using a manually assigned vector-field boundary outliner software tool [32]. The first axial cross-sectional area measurement was obtained from the midline articular space just above the tibial spine. The last measurement was obtained from the inferior aspects of the ankle, where all lower leg muscles had already vanished, leaving only their tendons. The tracing technique delimited the entire visible area of the muscle or tendon, excluding the peritendinous sheath. Subsequently, the software calculated the axial cross-sectional area [28–32, 35–38]. The maximum area (in square centimeters) of the muscles and tendons was obtained after three subsequent measurements along the three maximum subjectively observed areas of each structure, and the highest value was considered for statistical analysis. Muscle volumes were assessed by summing the volumes of the slices and the volumes of 2.4-cm gaps between the slices using a truncated cone formula (Figure 1) [32]. For all measurements, two independent raters, both physicians, one an orthopedic resident (Rater 1) and the other a radiologist (Rater 2), were trained independently by a senior radiologist. To compare the structural muscle and tendon properties between groups, all data were normalized to body mass to the power of 3/4 (m^3/4^). The power of 3/4 was chosen because allometric parameters that relate surfaces (e.g., muscle axial cross-sectional areas and volumes) to body mass are closer to 3/4 than to the 2/3 predicted by geometric similarity [39–41].

### 2.5. Statistical Analysis

The quantitative outcomes were expressed as mean and standard deviation. Normality was consistently checked using the Shapiro–Wilk test. The multivariate analysis of variance (MANOVA) model with Pillai's trace assessed the DPC and superficial posterior compartment muscle volumes, data considered multivariate parametric data (Henze–Zirkler test > 0.05) with homogeneity of variance–covariance matrices (Box′s *M*‐test > 0.001). The unpaired *t*-test assessed weight, body mass index (BMI), maximum tendon cross-sectional areas, maximum muscle axial cross-sectional areas, tibialis anterior volume, extensor digitorum longus volume, extensor hallucis longus volume, and FL volume. The intraclass correlation coefficient (ICC) accessed interrater reliability for all absolute records, and the Rater 1 measurements were considered for analysis purposes. The ICC was classified in the following manner: moderate (0.5–0.75), good (> 0.75–0.9), and excellent (> 0.9). Pearson's chi-square test was used to determine whether the categorical variables had an influence in the MTSS group. The effect sizes were calculated using as magnitude parameters the values described by Cohen [42]. Statistical significance was set at *p* < 0.05. No a priori power analyses were performed as no prior data were available at the time of study planning to calculate power in advance. All statistical analyses were performed using IBM SPSS Statistics software version 28.0 for Windows (SPSS Inc., Chicago, Illinois, United States).

## 3. Results

Our sample included 30 participants (mean age 32.3 ± 11.3 years) which were divided into two groups: MTSS group (*n* = 18; mean age 30.4 ± 12.4 years) and the control group (*n* = 12; mean age 35.2 ± 9.3 years) (Table 1). The ICC results showed reliability in the measurements of the lower leg (varying from 0.886 to 0.999).

**Figure 2:**
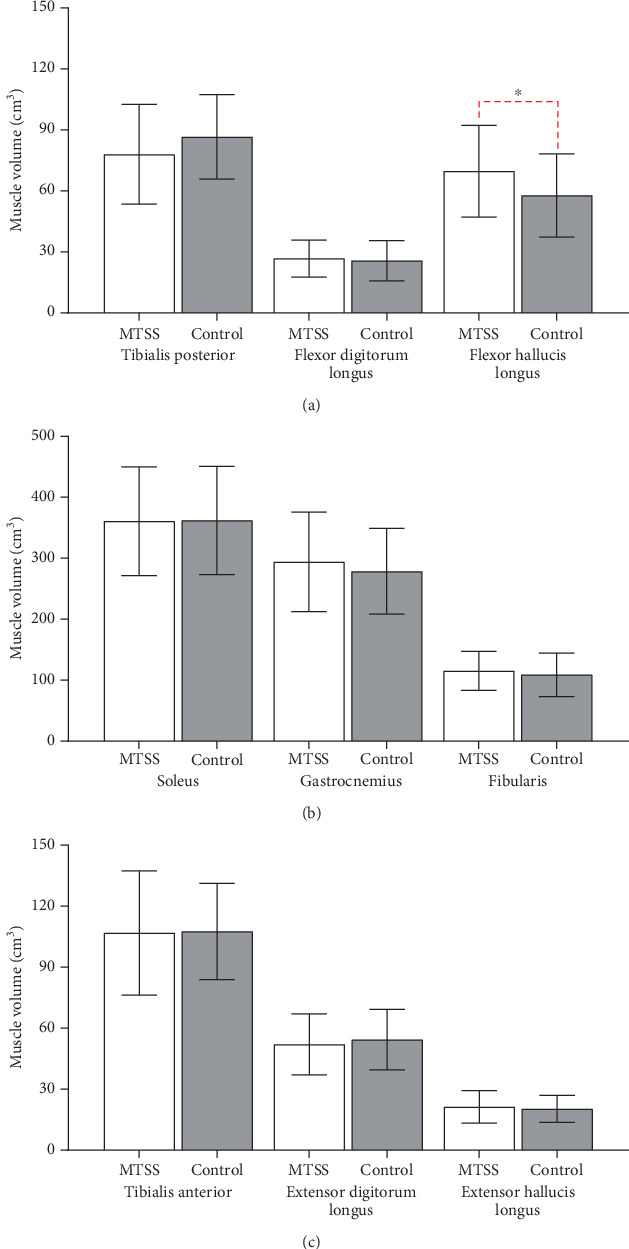
Bar graphs comparing (a) lower leg deep posterior compartment muscle volume, (b) lower leg superficial posterior and lateral compartment muscle volume, and (c) lower leg anterior compartment muscle volume. Red dotted loops indicate comparisons between muscle groups between MTSS standard deviation. MTSS = medial tibial stress syndrome group, control = control group, cm^3^ = cubic centimeter.

**Figure 3:**
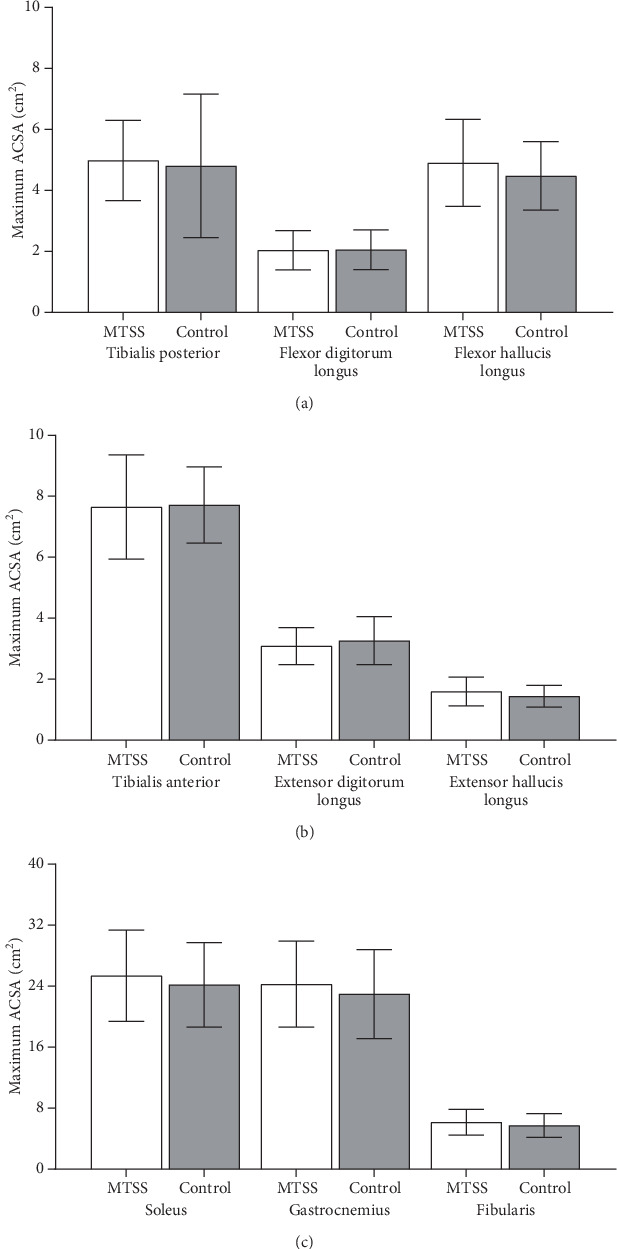
(a) Bar graphs comparing lower leg deep posterior compartment muscle maximum axial cross-sectional area (ACSA). (b) Bar graphs comparing anterior compartment muscle maximum axial cross-sectional area (ACSA). (c) Bar graphs comparing superficial posterior and lateral compartment muscle maximum axial cross-sectional area (ACSA). SD = standard deviation, MTSS = medial tibial stress syndrome group, control = control group, cm^2^ = square centimeter. There were no differences between groups (*p* > 0.05).

**Figure 4:**
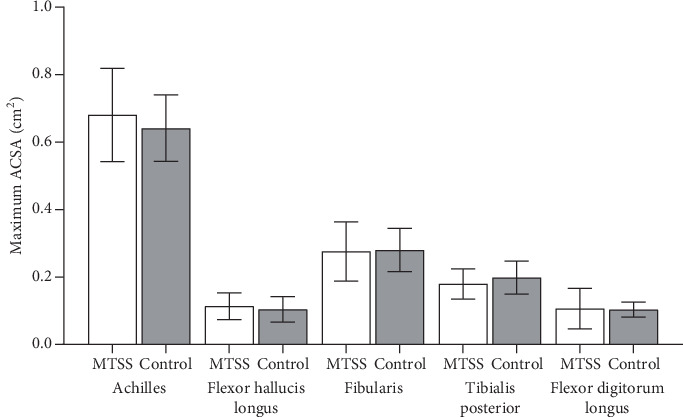
Bar graphs comparing lower leg tendon maximum axial cross-sectional area (ACSA) excluding anterior compartment tendons. SD = standard deviation, MTSS = medial tibial stress syndrome group, control = control group, cm^2^ = square centimeter. There were no differences between groups (*p* > 0.05).

The mean DPC was significantly greater in volume difference of 0.41 cm^3^/kg^3/4^ in the MTSS group (Pillai′s trace = 0.280; *Z* (3, 26) = 3.367; *p* = 0.034) compared to the control group (2.52% greater mean muscle volume). This difference was due to the FHL as shown by the subsequent univariate analysis of variance (ANOVA), which demonstrated 0.55 cm^3^/kg^3/4^ superior normalized volume in the MTSS group compared to the control group (*F* (1, 28) = 5.772; *p* = 0.023) (17.12% greater mean muscle volume). The model fit also stated that there was no difference in the volumes of the other DPC muscles (Figure 2), no differences between groups for the normalized volumes of the superficial posterior compartment muscles (Pillai′s trace = 0.101; *F* (6, 23) = 0.433; *p* = 0.101) (Figure 2) (Table 2), and no association of gender with MTSS in our study (*X*^2^(1) = 0.201; *p* = 0.654) (Table 1). No significant differences (*p* > 0.05) were found for throughout the independent *t*-tests for all other exploratory data (Tables 3 and 4) (Figures 2, 3, and 4).

## 4. Discussion

To our knowledge, this is the first study to quantify the leg muscle volumes and tendon axial cross-sectional areas of individuals with MTSS measured by MRI. Our results showed a greater normalized volume of the FHL muscle, which is in accordance with the possible tenting effect involving DPC that may cause MTSS [24]. Based on our results, it is possible to suggest that this increased volume should not directly impact the PTB, as this muscle does not share any origins with the tibia. However, it should directly impact the transverse intermuscular septum, which is conjoined with the tibial fascia in the PTB, thus creating the tenting effect as previously described [43]. Nevertheless, Saeki et al. proposed a different hypothesis, suggesting that there is no direct relation of the FHL to the tibial fascia. Instead, they proposed a possible increased action of the FHL to reduce load to the FDL, thereby alleviating pain caused by contraction stress of the FDL [25]. Several studies have demonstrated excessive pronation and a positive navicular drop test in runners with MTSS, indicating these as risk factors for stress-related injuries [18–21]. Other studies have claimed the relationship of the DPC muscles with excessive pronation and MTSS [24, 25, 44].

Bouché and Johnson proposed a pathomechanical model involving fascial traction of the PTB. They claimed that anatomical, pathological, diagnostic, and clinical findings were consistent with the origin of MTSS, describing the tibial fascia as inserted along the entire length of the medial crest of the tibia, with no other anatomical structure related to it. They associated MTSS cases with widespread PTB pain and proposed that heightened tibial fascia tension results from the eccentric contraction of leg flexor tendons, introducing the term “tenting effect” [24]. In the context of increased eccentric contractions of the deep flexors observed during conditions of excessive pronation, Saeki et al. proposed that athletes with a history of MTSS might employ a strategy to reduce medial tibial loading [25]. They demonstrated increased strength in the DPC muscles through a strength measurement study using an electric dynamometer [25]. Further prospective biomechanical studies are warranted to elucidate the causal relationship between DPC muscles and MTSS, particularly considering the superior volume of the FHL observed in our study.

A well-conducted umbrella review evaluates the effectiveness of lower extremity injury prevention programs based on physical exercises and strength training of the leg muscles. They clearly concluded that these exercises overwhelmingly reduce the chance of developing lesions in the lower limb [45]. Moreover, muscular fatigue is the core of one of the most studied hypotheses on the etiology and etiopathology of MTSS [21]. Nevertheless, several studies reported the anatomical findings of the lower leg muscle origins. They refuted the traction theory (tenting effect), as they expressed the absence of direct muscle insertions on the distal two-thirds of the PTB [12, 46, 47]. As we discussed, the structure directly related to all PTB length is the tibial fascia [48]. It is not possible to rule out the tenting effect theory based only on anatomical muscle direct insertion and origin based on our results, but rather than considering different etiology and etiopathology of MTSS.

Our study did not find an association between gender and MTSS, although existing literature indicates a predominance of females affected by this syndrome [6, 18–21]. Additionally, a history of lower limb musculoskeletal injuries is recognized as a risk factor for MTSS [4]. However, our study design excluded individuals with previous limb injuries to mitigate potential effects on morphometric parameters of muscles, ligaments, and tendons [49]. This exclusion is aimed at reducing bias in measurements, leading to more homogeneous groups with fewer significant differences. Another factor potentially contributing to fewer differences between groups is the convenience sampling of control participants. Although we selected patients with normal MRI exams of the lower leg and no pain in the PTB for the control group, they did experience pain in other regions of the leg not related to MTSS. In addition, MTSS participants showed a higher volume of the FHL muscle compared to controls, reinforcing the statistical significance of the observed differences amid a trend towards group homogeneity.

Several limitations were identified in this study. The retrospective, cross-sectional design limited our ability to establish causality. Additionally, the absence of an a priori sample size calculation may have resulted in an insufficient sample, potentially affecting the detection of differences in other DPC muscles. The inclusion of sedentary participants and the lack of standardized pre-exam physical activity instructions could introduce variability. However, the observed difference was specific to one muscle group and did not appear in others, suggesting minimal impact on the overall findings. Variability in MTSS symptom presentation and treatment protocols also represent potential confounding factors. Future studies with a prospective design, incorporating bilateral evaluation of the unaffected contralateral limb, could reduce the need for a separate control group, minimize confounders, and help clarify associations with DPC muscles.

## 5. Conclusion

The increased volume of the FHL muscle was observed in individuals with MTSS compared to the control group, along with no association between gender and MTSS. Further investigation on the relevance between muscle morphology and MTSS, as well as exploring additional risk factors, could be beneficial for improving patient outcomes.

## Figures and Tables

**Table 1:** Group characteristics and sports activity data.

	**MTSS**	**Normal**	**p**
BMI	24.44 ± 3.28	25.60 ± 3.01	0.257^b^
Age	30.39 ± 12.415	35.25 ± 9.26	0.337^b^
Side (left)	77.80%	58.30%	-
Gender (male)	50%	58.30%	0.654^c^
Physical activity^a^	94.40%	75%	-
Running^a^	72.20%	25%	0.011^c^
Strength training^a^	33.33%	33.3%	-
Soccer^a^	16.70%	0%	-
Swimming^a^	11.10%	0%	-
Cycling^a^	11.10%	16.70%	-
Equestrianism^a^	5.60%	0%	-
Sports court^a^	5.60%	8.30%	-

*Note:* Side, frequency of which leg was examined; physical activity, the practice of any physical activities; sports court, sports activities practiced indoor (e.g., basketball and volleyball); -, did not meet Pearson's chi-square minimum criteria. There were no significant differences between groups after post hoc analysis of Bonferroni (*p* < 0.01).

Abbreviations: BMI, body mass index; MTSS, medial tibial stress syndrome group; normal, normal group; *p*, *p* value.

^a^Practice of.

^b^Student's *t*-test.

^c^Pearson's chi-square test.

**Table 2:** MANOVA results. Comparisons between MTSS and control groups.

	**MTSS value**	**Control value**	**Shapiro–Wilk (** **p** **)**	**Levene (** **p** **)**	**p**	**Cohen ** **d**	**Effect sizes**
TP volume normalized	3.15	3.38	0.130	0.923	0.461	−0.278	Small
FDL volume normalized	1.08	0.98	0.175	0.967	0.463	0.277	Small
FHL volume normalized	2.76	2.21	0.589	0.922	0.023⁣^∗^	0.895	Large
GT volume normalized	11.73	10.78	0.293	0.963	0.248	0.432	Medium
SL volume normalized	14.26	13.98	0.805	0.463	0.599	0.554	Medium

*Note:* Volume normalization (cm^3^/kg^3/4^). MTSS value and control values in cm^3^/kg^3/4^.

Abbreviations: FDL, flexor digitorum longus; FHL, flexor hallucis longus; GT, gastrocnemius; SL, soleus; TP, tibialis posterior.

⁣^∗^There was significant difference of the normalized FHL volume between MTSS and control groups.

**Table 3:** Independent *t*-test results. Comparisons between MTSS and control groups.

	**MTSS value**	**Control value**	**Shapiro–Wilk (** **p** **)**	**Levene (** **p** **)**	**p**	**Cohen ** **d**	**Effect sizes**
BMI	24.44	25.59	0.845	0.415	0.440	−1.097	Large
FDL muscle maximum ACSA normalized	0.82	0.79	0.200	0.174	0.289	0.178	Small
FHL muscle maximum ACSA normalized	0.19	0.17	0.200	0.529	0.085	0.525	Medium
TP muscle maximum ACSA normalized	0.201	0.188	0.008	0.087	0.604	−0.196	Small
TA muscle maximum ACSA normalized	0.30	0.30	0.988	0.002	0.662	−0.165	Small
EDL muscle maximum ACSA normalized	0.124	0.126	0.307	0.888	0.766	−0.620	Medium
EHL muscle maximum ACSA normalized	0.064	0.055	0.154	0.056	0.130	−0.581	Medium
FL muscle maximum ACSA normalized	0.245	0.221	0.506	0.795	0.158	−0.541	Medium
SL muscle maximum ACSA normalized	1.015	0.936	0.017	0.726	0.113	−0.609	Medium
GT muscle maximum ACSA normalized	0.971	0.891	0.521	0.127	0.182	−0.510	Medium

*Note:* ACSA normalization (mm^2^/kg^3/4^); MTSS and control group values in mm^2^/kg^3/4^. There were no significant differences between groups.

Abbreviations: ACSA, axial cross-sectional area; EDL, extensor digitorum longus; EHL, extensor hallucis longus; FDL, flexor digitorum longus; FHL, flexor hallucis longus; FL, fibularis; GT, gastrocnemius; SL, soleus; TA, tibialis anterior; TP, tibialis posterior.

**Table 4:** Independent *t*-test results. Comparisons between MTSS and control groups.

	**MTSS value**	**Control value**	**Shapiro–Wilk (** **p** **)**	**Levene (** **p** **)**	**p**	**Cohen ** **d**	**Effect sizes**
TP tendon maximum ACSA normalized	0.0073	0.0077	0.487	0.938	0.405	−0.076	Small
FHL tendon maximum ACSA normalized	0.0045	0.0040	0.572	0.087	0.059	0.389	Small
AT tendon maximum ACSA normalized	0.0276	0.0253	0.464	0.629	0.260	−0.429	Small
FDL tendon maximum ACSA normalized	0.0043	0.0040	< 0.001	0.280	0.761	−0.114	Small
FL tendon maximum ACSA normalized	0.0112	0.0109	0.919	0.049	0.780	−0.835	Large

*Note:* ACSA normalization (mm^2^kg^3/4^); MTSS and control group values in mm^2^/kg^3/4^. There were no significant differences between groups.

Abbreviations: ACSA, axial cross-sectional area; AT, Achilles tendon; FDL, flexor digitorum longus; FHL, flexor hallucis longus; FL, fibularis.

## Data Availability

The data that support the findings of this study are available from the corresponding author upon reasonable request.
